# COVID-19 Risk Appears to Vary Across Different Alcoholic Beverages

**DOI:** 10.3389/fnut.2021.772700

**Published:** 2022-01-03

**Authors:** Xi-jian Dai, Liang Tan, Lina Ren, Yuan Shao, Weiqun Tao, Yongjun Wang

**Affiliations:** ^1^Shenzhen Mental Health Centre, Shenzhen Kangning Hospital, Shenzhen, China; ^2^Department of Neurosurgery, Southwest Hospital, The Third Military Medical University (Army Military Medical University), Chongqing, China

**Keywords:** COVID-19, mortality, alcohol consumption, prospective cohort, risk factor, SARS-CoV-2, UK Biobank, drinker

## Abstract

**Objectives:** To evaluate the associations of status, amount, and frequency of alcohol consumption across different alcoholic beverages with coronavirus disease 2019 (COVID-19) risk and associated mortality.

**Methods:** This study included 473,957 subjects, 16,559 of whom tested positive for COVID-19. Multivariate logistic regression analyses were used to evaluate the associations of alcohol consumption with COVID-19 risk and associated mortality. The non-linearity association between the amount of alcohol consumption and COVID-19 risk was evaluated by a generalized additive model.

**Results:** Subjects who consumed alcohol double above the guidelines had a higher risk of COVID-19 (1.12 [1.00, 1.25]). Consumption of red wine above or double above the guidelines played protective effects against the COVID-19. Consumption of beer and cider increased the COVID-19 risk, regardless of the frequency and amount of alcohol intake. Low-frequency of consumption of fortified wine (1–2 glasses/week) within guidelines had a protective effect against the COVID-19. High frequency of consumption of spirits (≥5 glasses/week) within guidelines increased the COVID-19 risk, whereas the high frequency of consumption of white wine and champagne above the guidelines decreased the COVID-19 risk. The generalized additive model showed an increased risk of COVID-19 with a greater number of alcohol consumption. Alcohol drinker status, frequency, amount, and subtypes of alcoholic beverages were not associated with COVID-19 associated mortality.

**Conclusions:** The COVID-19 risk appears to vary across different alcoholic beverage subtypes, frequency, and amount. Red wine, white wine, and champagne have chances to reduce the risk of COVID-19. Consumption of beer and cider and spirits and heavy drinking are not recommended during the epidemics. Public health guidance should focus on reducing the risk of COVID-19 by advocating healthy lifestyle habits and preferential policies among consumers of beer and cider and spirits.

## Introduction

Coronavirus disease 2019 (COVID-19) pandemic has revealed how readily viruses spread in our interconnected world. As of November 20, 2021, more than 257 million COVID-19 cases and 5.2 million deaths have been reported worldwide (https://www.worldometers.info/coronavirus/). During the pandemic, although epidemiological characteristics and risk factors (e.g., age, obesity, and lifestyle factors) of COVID-19 have been rapidly reported (1–5), the risk or protective factors for COVID-19 infection are largely unknown.

Adverse effects of alcohol consumption have been widely documented. The observed relationships between alcohol consumption and diseases are often non-linear, with low-to-moderate alcohol consumption being protective and heavy alcohol consumption being harmful (6, 7). In addition, evidence has suggested the protective effects of moderate alcohol consumption on cardiovascular events, such as coronary heart disease (8), myocardial infarction (9), and heart failure (10). Several cohort studies have pointed out that people who have light-to-moderate alcohol consumption survive longer than abstainers (11). However, the association of alcohol consumption with the risk of COVID-19 infection or pneumonia was inconsistent (12–15), and the associations of status, amount, and frequency of different alcoholic beverages with the risk of COVID-19 and associated mortality have not been systematically investigated. In the present large longitudinal observational study, we systematically investigated the associations of alcohol consumption (status, frequency, amount, and subtype of alcoholic beverages) with COVID-19 risk and associated mortality after adjusting for several confounders. We further examined the “dose” -response association between alcohol consumption and COVID-19 risk by drawing the risk trajectory of COVID-19 with different amounts of alcohol consumption.

## Materials and Methods

### Subjects

After removing the death data (*n* = 28,547) before November 31, 2019, the remaining data of 473,958 subjects (212,067 men, aged 69.3 ± 8.2 years; 261,890 women, aged 69.1 ± 8.0 years; one missing data) from 22 centers between March 2006 and December 2010 in the UK Biobank were analyzed. Confirmed COVID-19 infection was defined as at least one positive test result. We discovered that 77,217 subjects received COVID-19 test results up to July 26, 2021, and that 16,559 subjects (men, *n* = 7,802) were confirmed to have positive COVID-19 test results. The ethical approval was obtained from the North-West Multi-Center Research Ethics Committee (REC reference: 16/NW/0274).

### Exposure Measures

We analyzed the association of baseline alcohol consumption with COVID-19 risk and associated mortality by the status of an alcohol drinker, frequency of alcohol consumption, and weekly amounts of alcohol consumption. The status of an alcohol drinker was grouped into non-drinker, previous drinker, and current drinker. To assess the alcohol drinker status (Data-field ID 20117), subjects were asked to answer the “present/past status of alcohol drinker” with four responses: “Prefer not to answer,” “Never,” “Previous,” and “Current.” The frequency of alcohol consumption was divided into low frequency (<3 times a week), high frequency (≥3 times a week), and never (never and special occasions only). To assess the alcohol intake frequency (ID 1558), subjects were asked to answer the question “About how often do you drink alcohol” with seven responses: “Prefer not to answer,” “Never,” “Daily or almost daily,” “Three or four times a week,” “Once or twice a week,” “One to three times a month,” and “Special occasions only.”

The amount of alcohol consumption was quantified as the average weekly number of units of alcohol consumption, which was computed by summing the average weekly intake of red wine (ID 1568), champagne plus white wine (ID 1578), beer and cider (ID 1588), spirits (ID 1598), and fortified wine (ID 1608). For example, to assess the amount of weekly intake of red wine, subjects were asked to answer the question. “In an average WEEK, how many glasses of red wine would you drink? (There are six glasses in an average bottle)” with a response by an exact value. The amount of weekly intake level of alcohol consumption was converted into units for beer and cider (1 pint = 2 units), wines (1 standard glass = 2 units), and spirits (1 shot = 1 unit) (3), and was grouped into four categories: (1) non-drinker, previous drinker, or special occasions only; (2) within recommended guidelines: <14 UK units/week; (3) above recommended guidelines: ≥14 units/week and <28 units/week; and (4) 2-fold or more above the recommended guidelines: ≥28 units/week (16).

### Categories of Confounding Factors

Confounding factors were demographic variables (age, sex, education level, ethnicity, employment status, body mass index (BMI), overall health rating, usually walking pace, and Townsend deprivation) and severe illness (cerebrovascular diseases, hypertension, cardiovascular diseases, diabetes, respiratory disease, and cancer). Obesity was defined as a BMI > 30 kg/m^2^. Usually, the walking pace was grouped into three categories: (1) normal walking pace; (2) slow walking pace; and (3) fast walking pace.

### Statistical Analyses

Continuous variables are presented as mean ± SD, and categorical variables are presented as a number (percentage). We used unpaired *t*-tests and χ^2^ tests to compare differences between the groups where appropriate.

For the continuous variable of alcohol consumption, the linear relation between the amount of alcohol consumption and COVID-19 risk was evaluated by a generalized additive model. *P*-values for non-linearity *p* < 0.05 suggested evidence against the linearity assumption.

Multivariate logistic regression analysis was used to evaluate the associations between alcohol consumption and COVID-19 risk and associated mortality, and to determine the odds ratios (*OR*s) and 95% *CI*s after adjusting for age, sex, education, ethnicity, employment, BMI, overall health rating, Townsend, usually walking pace, cerebrovascular diseases, hypertension, cardiovascular diseases, diabetes, respiratory disease, cancer, alcohol drinker status, frequency of alcohol intake, amount of weekly intake level of alcohol consumption, red wine, champagne plus white wine, beer and cider, spirits, and fortified wine. Two-tailed *p* < 0.05 was considered significant.

## Results

### Sample Characteristics

The demographic characteristics of the study population are presented in Table 1. Subjects who were positive for COVID-19 had a lower education level (*p* < 0.001), a fewer white ethnicity (*p* < 0.001), a poor overall health rating, a higher prevalence of Townsend deprivation score (*p* < 0.001), and more comorbidities (*p* < 0.001) than those who were negative for COVID-19. Furthermore, they were less likely to be alcohol drinkers (*p* < 0.001), had a lower frequency of alcohol consumption (*p* < 0.001) and had a lower amount of alcohol consumption than those who were negative for COVID-19. Consistent findings were found in non-survival subjects compared with survival subjects among those subjects who were positive for COVID-19.

**Table 1:** Characteristics of UK Biobank cohort.

**Characteristics**	**COVID-19 infection**		***p*-value**	**COVID-19 related mortality**		***p*-value**
	**COVID-19 (*n* = 16,559)**	**Non-COVID-19 (*n* = 457,399)**		**Non-survival (*n* = 664)**	**Survival (*n* = 15,895)**	
Age to August 2021 (years), mean ± SD	66.3 ± 8.6	69.3 ± 8.1	<0.001	75.9 ± 5.7	65.9 ± 8.5	<0.001
Sex (male), N (%)	7,802 (47.1)	204,265 (44.7)	<0.001	442 (66.6)	7,360 (46.3)	<0.001
Education (degree), N (%)	4,059 (25.1)	150,588 (33.6)	<0.001	109 (17.1)	3,950 (25.4)	<0.001
Ethnicity (white), N (%)	14,764 (89.6)	430,564 (94.6)	<0.001	605 (91.8)	14,159 (89.5)	0.062
Obesity (BMI ≥30 kg/m^2^), N (%)	5,071 (30.9)	108,512 (23.9)	<0.001	283 (43.6)	4,788 (30.4)	<0.001
Overall health rating, N (%)			<0.001			<0.001
Excellent or good	11,269 (68.6)	344,120 (75.7)		326 (49.8)	10,943 (69.4)	
Fair or poor	5,151 (31.4)	110,402 (24.3)		329 (50.2)	4,822 (30.6)	
Employment (in paid), N (%)	10,945 (66.6)	267,164 (58.8)		176 (26.6)	10,769 (68.2)	<0.001
Townsend deprivation, mean ± SD	−0.7 ± 3.3	−1.4 ± 3.1	<0.001	−0.2 ± 3.4	−0.7 ± 3.3	<0.001
Usually walking pace, N (%)			<0.001			<0.001
Normal	8,869 (54.3)	239,183 (52.7)		369 (57.2)	8,500 (54.2)	
Slow	1,638 (10.0)	33,234 (7.3)		154 (23.9)	1,484 (9.5)	
Fast	5,834 (35.7)	181,323 (40.0)		122 (18.9)	5,712 (36.4)	
Alcohol drinker status, N (%)			<0.001			<0.001
Never	1,020 (6.2)	19,967 (4.4)		46 (7.0)	974 (6.2)	
Previous	668 (4.1)	15,464 (3.4)		47 (7.1)	621 (3.9)	
Current	14,805 (89.8)	420,504 (92.2)		566 (85.9)	14,239 (89.9)	
Frequency of alcohol intake, N (%)			<0.001			<0.001
Never and special occasions only	3,827 (23.2)	87,536 (19.2)		201 (30.4)	3,626 (22.9)	
Once a month-twice a week	6,557 (39.7)	169,333 (37.1)		228 (34.5)	6,329 (40.0)	
≥ 3 times a week	6,118 (37.1)	199,198 (43.7)		232 (35.1)	5,886 (37.2)	
Alcohol consumption (dosage), mean ± SD	14.2 ± 19.1	14.7 ± 17.8	0.001	13.7 ± 18.4	14.3 ± 19.1	0.5
Alcohol consumption, dosage			<0.001			0.03
Never drinker, previous-drinkers or special occasions only	5,880 (35.5)	139,140 (30.4)		269 (40.5)	5,611 (35.3)	
Within guidelines	4,228 (25.5)	128,376 (28.1)		145 (21.8)	4,083 (25.7)	
Above guidelines	3,539 (21.4)	107,437 (23.5)		135 (20.3)	3,404 (21.4)	
Double above the guidelines or more	2,912 (17.6)	82,446 (18.0)		115 (17.3)	2,797 (17.6)	
Red wine drinkers, N (%)			<0.001			0.005
Non-drinkers	10,366 (62.6)	244,332 (53.4)		455 (68.5)	9,911 (62.4)	
1–2 glasses/week	1,989 (12.0)	66,151 (14.5)		60 (9.0)	1,929 (12.1)	
3–4 glasses/week	1,514 (9.1)	50,979 (11.1)		61 (9.2)	1,453 (9.1)	
≥ 5 glasses/week	2,690 (16.2)	95,937 (21.0)		88 (13.3)	2,602 (16.4)	
White wine and champagne drinkers			<0.001			<0.001
Non-drinkers	11,271 (68.1)	280,909 (61.4)		501 (75.5)	10,770 (67.8)	
1–2 glasses/week	2,232 (13.5)	75,360 (16.5)		71 (10.7)	2,161 (13.6)	
3–4 glasses/week	1,198 (7.2)	41,523 (9.1)		39 (5.9)	1,159 (7.3)	
≥ 5 glasses/week	1,858 (11.2)	59,607 (13.0)		53 (8.0)	1,805 (11.4)	
Beer and cider drinkers, N (%)			<0.001			0.026
Non-drinkers	10,122 (61.1)	291,031 (63.6)		393 (59.2)	9,729 (61.2)	
1–2 glasses/week	2,217 (13.4)	70,897 (15.5)		72 (10.8)	2,145 (13.5)	
3–4 glasses/week	1,269 (7.7)	32,696 (7.1)		61 (9.2)	1,208 (7.6)	
≥ 5 glasses/week	2,951 (17.8)	62,775 (13.7)		138 (20.8)	2,813 (17.7)	
Spirits drinkers, N (%)			<0.001			0.1
Non-drinkers	12,587 (76.0)	338,828 (74.1)		480 (72.3)	12,107 (76.2)	
1–2 glasses/week	1,835 (11.1)	61,352 (13.4)		81 (12.2)	1,754 (11.0)	
3–4 glasses/week	842 (5.1)	24,040 (5.3)		39 (5.9)	803 (5.1)	
≥ 5 glasses/week	1,295 (7.8)	33,179 (7.3)		64 (9.6)	1,231 (7.7)	
Fortified wine drinkers, N (%)			<0.001			0.2
Non-drinkers	15,774 (95.3)	425,258 (93.0)		630 (94.9)	15,144 (95.3)	
1–2 glasses/week	575 (3.5)	24,524 (5.4)		20 (3.0)	555 (3.5)	
3–4 glasses/week	106 (0.6)	4,275 (0.9)		8 (1.2)	98 (0.6)	
≥ 5 glasses/week	104 (0.6)	3,342 (0.7)		6 (0.9)	98 (0.6)	
Cerebrovascular diseases, N (%)	489 (3.0)	8,773 (1.9)	<0.001	69 (10.4)	420 (2.6)	<0.001
Hypertension, N (%)	4,043 (24.4)	96,140 (21.0)	<0.001	363 (54.7)	3,680 (23.2)	<0.001
Cardiovascular diseases, N (%)	1,358 (8.2)	30,985 (6.8)	<0.001	153 (23.0)	1,205 (7.6)	<0.001
Diabetes, N (%)	1,424 (8.6)	24,830 (5.4)	<0.001	171 (25.8)	1,253 (7.9)	<0.001
Respiratory disease, N (%)	3,397 (20.5)	72,131 (15.8)	<0.001	249 (37.5)	3,148 (19.8)	<0.001
Cancer, N (%)	1,865 (11.3)	58,892 (12.9)	<0.001	139 (20.9)	1,726 (10.9)	<0.001

### Association Between Alcohol Consumption and COVID-19 Risk

**Figure 1:**
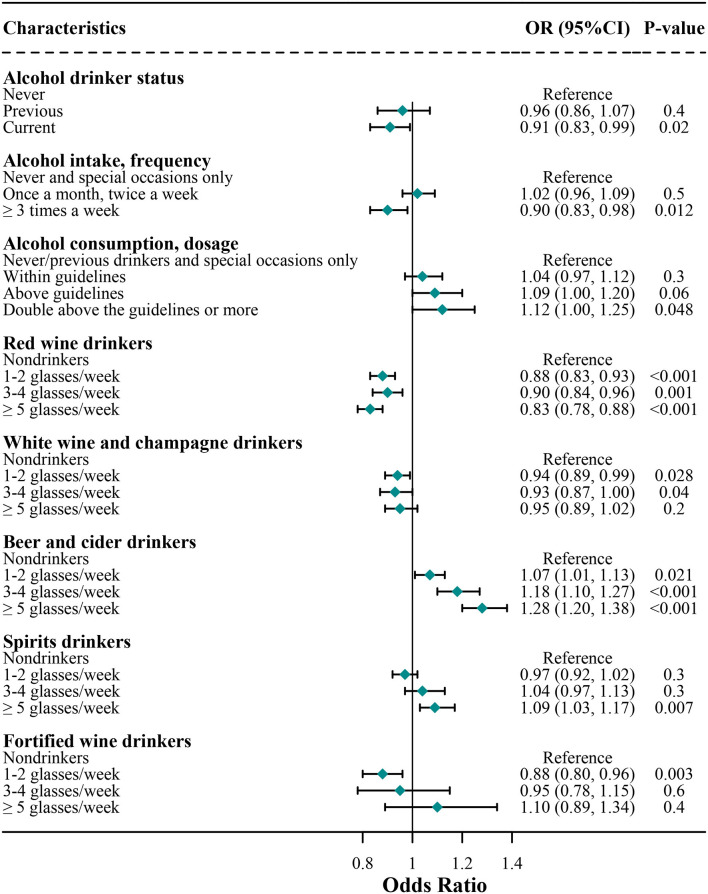
Association of alcohol consumption with risk of coronavirus disease 2019 (COVID-19) infection. Logistic regression analysis adjusted for age, sex, education, ethnicity, employment, body mass index (BMI), overall health rating, Townsend, usually walking pace, cerebrovascular diseases, hypertension, cardiovascular diseases, diabetes, respiratory disease, cancer, amount of weekly intake level of alcohol consumption, red wine, champagne plus white wine, beer and cider, spirits, and fortified wine.

The associations of alcohol consumption with COVID-19 risk are shown in Figure 1. In the final multivariate model, consumers who were current drinkers had a lower risk of developing COVID-19 compared with non-drinkers (*OR* [95% *CI*], 0.91 [0.83, 0.99]), but the protective effect was not significant for previous drinkers (0.96 [0.86, 1.07]). Furthermore, subjects who usually consumed alcohol at a high frequency had a 10% lower risk (0.90 [0.83, 0.98]) of developing COVID-19 compared with non-drinkers, but the protective effect was not significant for those subjects who usually consumed alcohol at a low frequency (1.02 [0.96, 1.09]). Subjects who usually consumed alcohol within guidelines were not associated with COVID-19 risk (1.04 [0.97, 1.12]) compared with non-drinkers; however, those consumers above guidelines had a tendency of a higher risk of COVID-19 (1.09 [1.00, 1.20]), and those consumers double above the guidelines or more had a 12% higher risk of COVID-19 (1.12 [1.00, 1.25]) compared with non-drinkers.

### Associations of Different Subtypes of Alcoholic Beverage With COVID-19 Risk

As shown in Figure 1, compared with non-drinkers, the COVID-19 risk was 10–17% lower in red wine consumers (1–2 glasses/week, 0.88 [0.83, 0.93]; 3–4 glasses/week, 0.90 [0.84, 0.96]; ≥ 5 glasses/week, 0.83 [0.78, 0.88]) regardless of the amount of red wine and 7–8% lower in white wine and champagne consumers (1–2 glasses/week, 0.94 [0.89, 0.99]; 3–4 glasses/week, 0.93 [0.87, 1.00]), but the protective effect was not significant when the amount of white wine and champagne was above 5 glasses/week (0.95 [0.89, 1.02]). Furthermore, compared with non-drinkers, fortified wine consumers of 1–2 glasses per week were associated with a 12% lower risk of COVID-19 (0.88 [0.80, 0.96]), whereas the consumption of a higher amount of fortified wine was not associated with lower COVID-19 risks (3–4 glasses/week, 0.95 [0.78, 1.15]; ≥ 5 glasses/week, 1.10 [0.89, 1.34]).

Compared with non-drinkers, the average consumption of 1–4 glasses/week of spirits was not significantly associated with COVID-19 risk among spirits consumers (1–2 glasses/week, 0.97 [0.92, 1.02]; 3–4 glasses/week, 1.04 [0.97, 1.13]); however, consumption of a higher number of spirits increased the risk of COVID-19 among spirits consumers (1.09 [1.03, 1.17]). Compared with non-drinkers, consumers of beer and cider had 7–28% higher risks of COVID-19 (1–2 glasses/week, 1.07 [1.01, 1.13]; 3–4 glasses/week, 1.18 [1.10, 1.27]; ≥5 glasses/week, 1.28 [1.20, 1.38]), regardless of the amount of beer and cider; that is, a higher amount of beer and cider corresponds to a higher COVID-19 risk.

### Sensitivity Analysis of the Association Between Alcohol Consumption and COVID-19 Risk

**Figure 2:**
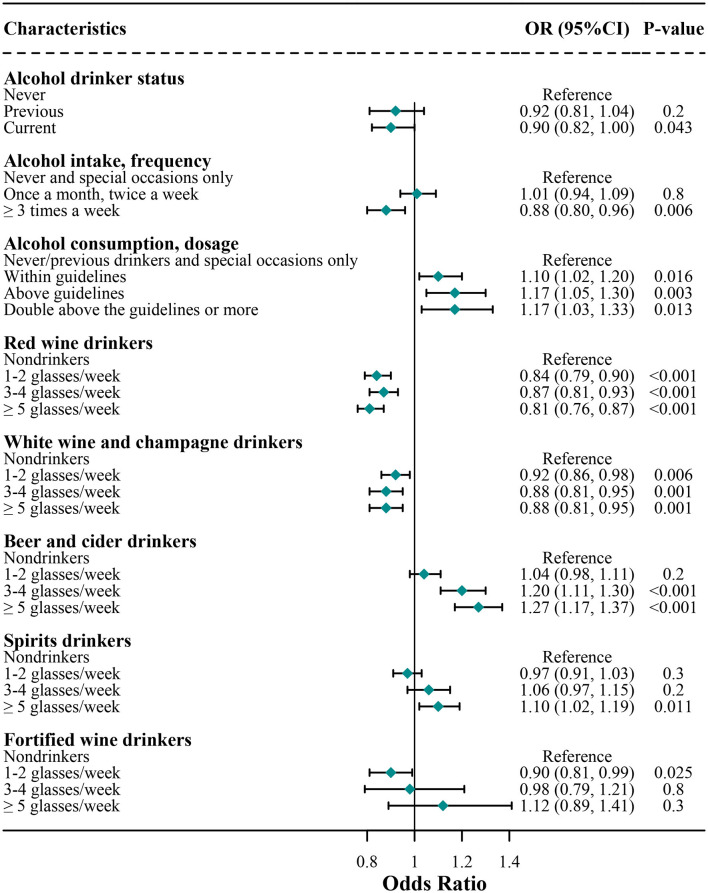
Sensitivity analysis for association of alcohol consumption with COVID-19 risk. Logistic regression analysis adjusted for age, sex, education, ethnicity, employment, BMI, overall health rating, Townsend, usually walking pace, cerebrovascular diseases, hypertension, cardiovascular diseases, diabetes, respiratory disease, cancer, amount of weekly intake level of alcohol consumption, red wine, champagne plus white wine, beer and cider, spirits, and fortified wine.

We further analyzed the association of alcohol consumption with COVID-19 risk among those subjects who have reported the COVID-19 testing results (Figure 2). We found that the results were highly similar to the findings as shown in Figure 1 where the subjects who did not report the COVID-19 testing results were deemed to have a negative COVID-19 testing result. Specially, additional findings of the sensitivity analysis showed that compared with non-drinkers, alcohol consumers had higher risks of COVID-19, regardless of within or above the guidelines (within guidelines, 1.10 [1.02, 1.20]; above guidelines, 1.17 [1.05, 1.30]; and double above the guidelines or more, 1.17 [1.03, 1.33]).

### Stratification Analysis

We further examined the association of different subtypes of alcoholic beverages with COVID-19 risk, separated by frequency of alcohol intake (Table 2) and amount of alcohol consumption (Table 3), respectively.

**Table 2:** Odds ratios (ORs) and 95% CIs for the association between alcohol consumption and coronavirus disease 2019 (COVID-19) risk, separated by frequency of alcohol intake.

**Variables (a)**	**Frequency of alcohol intake, OR (95% Cl)**			
	**Once a month-twice/week**	***p***	**≥ 3 times/week**	***p***
**Alcohol consumption, dosage**
Never drinker, previous-drinkers or special occasions only	Reference		Reference	
Within guidelines	1.01 (0.92–1.11)	0.8	0.96 (0.62–1.49)	0.9
Above guidelines	1.06 (0.91–1.24)	0.4	1.00 (0.64–1.54)	1.0
Double above the guidelines or more	1.07 (0.85–1.35)	0.6	1.04 (0.67–1.62)	0.9
**Red wine drinkers**
Nondrinkers	Reference		Reference	
1–2 glasses/week	0.89 (0.82–0.97)	0.005	0.88 (0.81–0.97)	0.006
3–4 glasses/week	0.90 (0.82–1.00)	0.044	0.90 (0.83–0.99)	0.023
≥ 5 glasses/week	0.82 (0.71–0.93)	0.003	0.83 (0.77–0.89)	<0.001
**White wine and champagne drinkers**
Non-drinkers	Reference		Reference	
1–2 glasses/week	0.95 (0.88–1.02)	0.2	0.93 (0.86–1.01)	0.069
3–4 glasses/week	0.94 (0.84–1.05)	0.3	0.91 (0.83–0.99)	0.037
≥ 5 glasses/week	1.06 (0.92–1.22)	0.4	0.91 (0.85–0.99)	0.019
**Beer and cider drinkers**
Non-drinkers	Reference		Reference	
1–2 glasses/week	1.11 (1.03–1.21)	0.01	1.06 (0.98–1.15)	0.2
3–4 glasses/week	1.24 (1.11–1.39)	<0.001	1.18 (1.07–1.31)	0.001
≥ 5 glasses/week	1.34 (1.17–1.53)	<0.001	1.31 (1.20–1.44)	<0.001
**Spirits drinkers**
Non-drinkers	Reference		Reference	
1–2 glasses/week	0.99 (0.91–1.07)	0.8	0.97 (0.90–1.04)	0.4
3–4 glasses/week	1.09 (0.97–1.23)	0.1	1.02 (0.92–1.12)	0.7
≥ 5 glasses/week	1.11 (0.97–1.26)	0.1	1.09 (1.01–1.17)	0.033
**Fortified wine drinkers**
Non-drinkers	Reference		Reference	
1–2 glasses/week	0.80 (0.69–0.92)	0.002	0.93 (0.83–1.04)	0.2
3–4 glasses/week	0.94 (0.65–1.35)	0.7	0.94 (0.74–1.19)	0.6
≥ 5 glasses/week	1.15 (0.70–1.89)	0.6	1.07 (0.86–1.34)	0.5

*Logistic regression analysis adjusted for age, sex, education, ethnicity, employment, body mass index (BMI), overall health rating, Townsend, usually walking pace, cerebrovascular diseases, hypertension, cardiovascular diseases, diabetes, respiratory disease, cancer, amount of weekly intake level of alcohol consumption, red wine, champagne plus white wine, beer and cider, spirits, and fortified wine*.

**Table 3:** Odds ratios and 95% CIs for the association between alcohol consumption and COVID-19 risk, separated by amount of alcohol consumption.

**Variables (b)**	**Alcohol consumption, dosage**					
	**Within guidelines**	***p*-value**	**Above guidelines**	***p*-value**	**Double above the guidelines or more**	***p*-value**
**Red wine drinkers**						
Non-drinkers	Reference		Reference		Reference	
1–2 glasses/week	0.90 (0.83–0.97)	0.01	0.80 (0.72–0.90)	<0.001	0.96 (0.82–1.12)	0.6
3–4 glasses/week	0.94 (0.85–1.04)	0.2	0.78 (0.70–0.88)	<0.001	0.93 (0.80–1.09)	0.4
≥ 5 glasses/week	0.86 (0.73–1.02)	0.1	0.71 (0.63–0.80)	<0.001	0.84 (0.77–0.93)	<0.001
**White wine and champagne drinkers**						
Non-drinkers	Reference		Reference		Reference	
1–2 glasses/week	0.94 (0.87–1.01)	0.1	0.96 (0.87–1.06)	0.4	0.91 (0.79–1.04)	0.2
3–4 glasses/week	0.91 (0.81–1.02)	0.1	0.89 (0.79–1.01)	0.063	1.00 (0.87–1.15)	1.0
≥ 5 glasses/week	0.92 (0.77–1.10)	0.4	0.84 (0.74–0.96)	0.01	0.98 (0.89–1.08)	0.7
**Beer and cider drinkers**						
Non-drinkers	Reference		Reference		Reference	
1–2 glasses/week	1.10 (1.01–1.20)	0.034	1.16 (1.05–1.29)	0.004	1.03 (0.89–1.18)	0.7
3–4 glasses/week	1.26 (1.11–1.42)	<0.001	1.22 (1.07–1.39)	0.003	1.18 (1.01–1.39)	0.035
≥ 5 glasses/week	1.21 (1.02–1.44)	0.026	1.30 (1.13–1.50)	<0.001	1.35 (1.19–1.53)	<0.001
**Spirits drinkers**						
Non-drinkers	Reference		Reference		Reference	
1–2 glasses/week	1.01 (0.93–1.10)	0.8	0.95 (0.87–1.04)	0.3	0.92 (0.82–1.04)	0.2
3–4 glasses/week	1.15 (1.01–1.30)	0.036	0.99 (0.87–1.12)	0.8	0.95 (0.82–1.10)	0.5
≥ 5 glasses/week	1.22 (1.06–1.41)	0.007	1.07 (0.95–1.20)	0.3	0.99 (0.89–1.09)	0.8
**Fortified wine drinkers**						
Non-drinkers	Reference		Reference		Reference	
1–2 glasses/week	0.79 (0.68–0.91)	0.002	0.95 (0.82–1.09)	0.5	0.90 (0.76–1.08)	0.3
3–4 glasses/week	1.01 (0.68–1.50)	1.0	0.86 (0.63–1.17)	0.3	0.99 (0.71–1.39)	1.0
≥ 5 glasses/week	0.59 (0.19–1.86)	0.4	1.05 (0.74–1.48)	0.8	1.13 (0.87–1.47)	0.4

*Logistic regression analysis adjusted for age, sex, education, ethnicity, employment, BMI, overall health rating, Townsend, usually walking pace, cerebrovascular diseases, hypertension, cardiovascular diseases, diabetes, respiratory disease, cancer, amount of weekly intake level of alcohol consumption, red wine, champagne plus white wine, beer and cider, spirits, and fortified wine*.

When stratifying our analysis by frequency of alcohol intake, we found that, among the subjects who usually reported consumption of alcohol at a low frequency, consumption of red wine regardless of amount (1–2 glasses/week, 0.89 [0.82, 0.97]; 3–4 glasses/week, 0.90 [0.82, 1.00]; and ≥ 5 glasses/week, 0.82 [0.71, 0.93]) and consumption of fortified wine 1–2 glasses/week (0.80 [0.69, 0.92]) were identified as protective factors against the COVID-19 contributing to a decreased risk of COVID-19. In contrast, the beer and cider, regardless of amount (1–2 glasses/week, 1.11 [1.03, 1.21]; 3–4 glasses/week, 1.24 [1.11, 1.39]; and ≥ 5 glasses/week, 1.34 [1.17, 1.53]), were identified as risk factors for COVID-19 compared with non-drinkers. Among those subjects who usually reported consumption of alcohol at a high frequency, the lower risk of red wine and the higher risk of beer and cider for COVID-19 were still significant, but the protective role of the fortified wine 1–2 glasses/week was not significant. Furthermore, the consumption of white wine and champagne (3–4 glasses/week, 0.91 [0.83, 0.99]; and ≥ 5 glasses/week, 0.91 [0.85, 0.99]) with a lower risk for COVID-19 and consumption of spirits (1.09 [1.01, 1.17]) with a higher risk were found.

When stratifying our analysis by the amount of alcohol consumption, we found that, among subjects who usually reported consumption of alcohol within the guidelines, the consumption of red wine 1–2 glasses/week (0.90 [0.83, 0.97]) and fortified wine 1–2 glasses/week (0.79 [0.68, 0.91]) was associated with lower risks of COVID-19, whereas the consumption of beer and cider regardless of amount (1–2 glasses/week, 1.10 [1.01, 1.20]; 3–4 glasses/week, 1.26 [1.11, 1.42]; and ≥5 glasses/week, 1.21 [1.02, 1.44]) and spirits above 3 glasses/week (3-4 glasses/week, 1.15 [1.01, 1.30]; and ≥ 5 glasses/week, 1.22 [1.06, 1.41]) were associated with higher risks of COVID-19 compared with non-drinkers. Among subjects who usually reported consumption of alcohol above the guidelines, the higher risk of beer and cider for COVID-19 was still significant, but the higher risk of spirits and lower risk of fortified wine were not significant. Specially, the protective role of the red wine was significant regardless of the amount of red wine among subjects who usually reported consumption of alcohol 1–2-fold above the guidelines. For those subjects who usually reported consumption of alcohol double above the guidelines, only consumption of red wine above 5 glasses/week decreased the risk of COVID-19 (0.84 [0.77, 0.93]).

### Associations of Alcohol Consumption With COVID-19 Associated Mortality

The association of alcohol consumption with the risk of COVID-19 associated mortality is shown in Supplementary Figure 1. Alcohol drinker status, frequency and amount of alcohol consumption, and all subtypes of alcoholic beverage were not associated with COVID-19 associated mortality.

### Non-linear Associations of Alcohol Consumption With COVID-19

**Figure 3:**
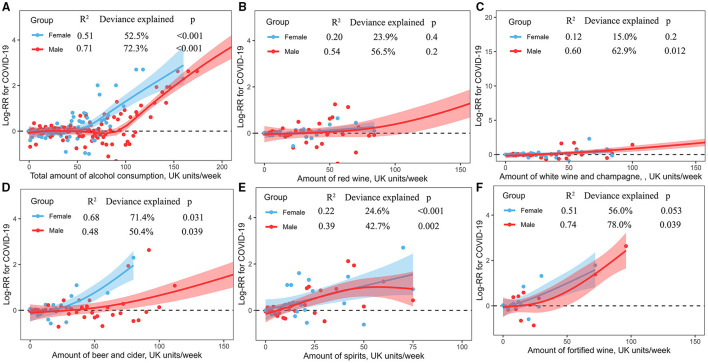
**(A–F)** Non-linear associations between the amount of alcohol consumption and COVID-19 risk.

The dose-response associations between the amount of alcohol consumption and COVID-19 risk are shown in Figure 3. Total amount of alcohol consumption showed a curvilinear J-shaped correlation with the risk of COVID-19 among male (*R*^2^ = 0.71, deviance explained = 72.3%, *p* < 0.001) and female (*R*^2^ = 0.51, deviance explained = 52.5%, *p* < 0.001) consumers, respectively, where female consumers had a higher risk of COVID-19 than male consumers (Figure 3A). Male and female consumers showed an overlap linear increase of COVID-19 risk with the amount of consumption of red wine (Figure 3B) and white wine and champagne (Figure 3C), respectively. The consumption of beer and cider had a curvilinear correlation with the risk of COVID-19 among male (*R*^2^ = 0.48, deviance explained = 50.4%, *p* = 0.039) and female (*R*^2^ = 0.68, deviance explained = 71.4%, *p* = 0.031) consumers, respectively, where alcohol consumers had an increased risk with a greater number of consumptions of beer and cider, and female consumers had a higher risk of COVID-19 than male consumers (Figure 3D). Male and female consumers showed a non-linear increase of COVID-19 risk with the amount of consumption of spirits (Figure 3E) and fortified (Figure 3F), respectively.

## Discussion

In this prospective study of a large sized UK Biobank cohort, we documented seven novel findings. First, we identified some independent protective and risk factors for COVID-19. The protective factors included current alcohol drinker status, high frequency of alcohol consumption, red wine, white wine, and champagne, and 1–2 glasses/week fortified wine; the risk factors included alcohol consumption within and above the guidelines, beer and cider (regardless of amount), and spirits (≥5 glasses/week). Second, the protective effect of red wine for COVID-19 was significant regardless of the frequency of alcohol intake, but it only played the protective effect when subjects consumed alcohol above or double above the guidelines. Third, the consumption of beer and cider increased the risk of COVID-19, regardless of the frequency and amount (within or above the guidelines) of alcohol intake. Fourth, low frequency of consumption of fortified wine (1–2 glasses/week) had a protective effect when subjects consumed alcohol within the guidelines. Fifth, a high frequency of consumption of spirits (≥5 glasses/week) within the guidelines was associated with a higher risk of COVID-19, whereas a high frequency of consumption of white wine and champagne above the guidelines was associated with a lower risk of COVID-19. Sixth, the dose-response associations between the amount of alcohol consumption and the risk of COVID-19 showed an increased risk of COVID-19 with a greater number of alcohol consumption. Seventh, alcohol drinker status, frequency and amount of alcohol consumption, and all subtypes of alcoholic beverage were not associated with COVID-19 associated mortality.

The most striking and interesting finding for COVID-19 infection was the recommended intake of different alcoholic beverages. The protective association of low-to-moderate alcohol consumption with a lower risk of cataracts (17), a lower risk of myocardial infarction (6), and a better cognitive function (18) have been reported. In our study, the consumption of different alcoholic beverages was associated with different chances of COVID-19 infection. It is worth noting that red wine is recommended for all adults because it was associated with a lower risk of COVID-19 when subjects usually consumed alcohol above or double above the guidelines. The maximum recommended intake of fortified wine is 2 glasses/week within the guidelines, which may have chances to reduce the COVID-19 risk. The consumption of beer and cider are not recommended, regardless of frequency and amount of alcohol consumption. Furthermore, heavy drinking is not recommended for all alcoholic beverages. Therefore, the COVID-19 risk appears to vary across different alcoholic beverages, frequency and amount of alcohol consumption.

In addition, we evaluated the dose-response associations between the amount of alcohol consumption and the COVID-19 risk. We noted a non-linear J-shaped or linearity increase of the COVID-19 risk with the weekly amount of alcohol consumption across different substyles of alcoholic beverages. Studies have shown that long-term alcohol abuse and acute binge drinking are associated with immunosuppression and increased susceptibility to both bacterial and viral infections (19, 20). Therefore, heavy alcohol consumption was associated with a higher risk of COVID-19. However, in our dataset, only a few participants reported heavy alcohol consumption, which may be reflective of the healthier nature of study participants. The potential association of heavy alcohol consumption with COVID-19 and associated mortality should be further explored in a larger sample sized cohort (although ascertaining the prospective effect of heavy alcohol consumption would be challenging because heavy drinkers would be unlikely to volunteer for such studies).

Hamer et al. found a lower risk of COVID-19 for drinkers relative to non-drinkers if the amount of alcohol consumption was below the recommended guideline (3). Our results showed that some subtypes of alcoholic beverages (e.g., wine) themselves played protective roles against the COVID-19, whereas some subtypes increased the COVID-19 risk (e.g., beer and cider, and spirits). There are several possible explanations for these findings. Polyphenols, which are present in varying degrees in alcoholic beverages, have antioxidant properties, particularly in wines among which red wine has the highest concentrations of phenolic compounds (21). Studies have shown that wines exhibit beneficial properties which are independent of the presence of alcohol, and should be attributed to their polyphenolic contents (22, 23). Red wine provides additional benefits to other alcoholic beverages probably due to its higher polyphenolic content, by decreasing blood pressure, inhibiting the oxidation of low-density lipoprotein particles and other favorable effects on the cellular redox state, improving endothelial function, inhibiting platelet aggregation, reducing inflammation and cell adhesion, and activating proteins that prevent cell death (21). Polyphenols could inhabit the effects of several types of virus, such as Epstein-Barr virus (24, 25), enterovirus (26, 27), herpes simplex virus (HSV) (28), influenza virus (29), and other virus causing respiratory tract-related infections (30, 31). These findings support the notion of the strong beneficial properties of red wine against the COVID-19 risk.

Spirits had the highest alcohol concentration and the lowest polyphenolic concentration. We discovered a higher risk of spirits for COVID-19. Sanja Radonjić et al. have reported the differences between wine and beer in the presence and the concentrations of phenolic substances (32). They found that chalcones and flavanones were found only in beer, whereas other polyphenol classes (e.g., stilbenes, proanthocyanidins, and resveratrol) are found mainly in wine and champagne. These findings may suggest that the specific class of polyphenolic constituents may be responsible for the beneficial effect of alcoholic beverages on COVID-19 events, and not the alcohol concentration. The concentration of polyphenol may greatly vary in different beer productions because it is related to the specific ingredients (33, 34). The main raw materials used in the production of beer are malt and hops, which are also the main source of polyphenols. Previous studies have suggested that moderate consumption of beer has a positive effect on some biomarkers associated with human health (35, 36), which may be associated with the nutrient effect of the polyphenols in the beer. However, the beer was identified as an independent risk factor for COVID-19 in our study. In our study, the ingredients and concentration of polyphenol of alcoholic beverage subtypes were not considered, and a study with more details about the ingredients and concentration of polyphenol is needed in the future.

Severe acute respiratory syndrome coronavirus (SARS-CoV)-2 not only attacks the respiratory system but also the cardiovascular system (37). The SARS-CoV-2-induced endothelial disruption and vascular thrombosis in the lungs may be the major pathological process of COVID-19 (38, 39). A meta-analysis involving 63 studies has indicated that moderate alcohol consumption had beneficial effects on the cardiovascular system (35). Also, the peri-infarct inflammatory infiltration following myocardial infarction was positively modulated by white wine (40). We speculated that the mechanisms responsible for the beneficial effects of moderate alcohol consumption on the cardiovascular system against the COVID-19 could be explained by increased changes in the plasma antioxidant activity (41) and reductions in the level of low-density lipoprotein (LDL) cholesterol (42–44).

The major strengths of this study are the prospective, large United Kingdom population-based cohort, dose-response associations of alcohol consumption with COVID-19 risk, and a focus on the subgroup analyses for different alcoholic beverages. However, there are several limitations that should be addressed. First, subjects in the UK Biobank have a restricted age range, and therefore our data could not represent the whole population (2, 5). Second, alcohol consumption was measured at baseline, and we did not know about potential changes during the COVID-19 pandemic. However, our study demonstrated that long-term drinking habits were associated with COVID-19 risk and associated mortality, and therefore our results will not largely be affected by the short-term changes of drinking behavior by the pandemic and associated lockdowns. Third, recruiting heavy drinkers to test different alcoholic beverages for dose–response analyses is difficult. Fourth, whether these findings are suitable for the young population and Asian are unknown. Fifth, the ingredients and the concentration of polyphenol of alcoholic beverage subtypes were not considered.

## Conclusions

In conclusion, our study evaluated specific protective and risk factors across different alcoholic beverage subtypes for COVID-19 and found that the COVID-19 risk appears to vary across different subtypes, amounts, and frequency of alcoholic beverages. Our study suggests that subjects who usually consumed red wine and white wine and champagne above guidelines, and sometimes consumed 1–2 glasses/week fortified within the guidelines appear to have chances to reduce the risk of COVID-19. The consumption of beer and cider are not recommended regardless of frequency and amount of alcohol consumption, which increased the risk of COVID-19. Furthermore, heavy drinking is not recommended for all alcoholic beverages. Public health guidance should focus on reducing the risk of COVID-19 by advocating healthy lifestyle habits and preferential policies among consumers of beer and cider and spirits.

## Data Availability Statement

The original contributions presented in the study are included in the article/Supplementary Material, further inquiries can be directed to the corresponding author/s.

## Ethics Statement

The studies involving human participants were reviewed and approved by the North West Multi-Centre Research Ethics Committee (REC reference: 16/NW/0274). The patients/participants provided their written informed consent to participate in this study.

## Author Contributions

X-jD and YW had the idea for and designed this study, had full access to all the data in this study, take responsibility for the integrity of the data and the accuracy of the data analysis, critically revised the manuscript for important intellectual content, and gave final approval for the version to be published. X-jD and LT drafted the manuscript and did the analysis. YW, LR, YS, and WT take the responsibility for double check of the data analysis. All authors agree to be accountable for all aspects of the work in ensuring that questions related to the accuracy or integrity of any part of the work are appropriately resolved.

## Funding

This research has been conducted using the UK Biobank Resource under Application Number 75732. This work was supported by the Guangdong Basic and Applied Basic Research Foundation (Grant No. 2020A1515011469), and the Sanming Project of Medicine in Shenzhen (Grant No. SZSM201812052).

## Conflict of Interest

The authors declare that the research was conducted in the absence of any commercial or financial relationships that could be construed as a potential conflict of interest.

## Publisher's Note

All claims expressed in this article are solely those of the authors and do not necessarily represent those of their affiliated organizations, or those of the publisher, the editors and the reviewers. Any product that may be evaluated in this article, or claim that may be made by its manufacturer, is not guaranteed or endorsed by the publisher.
