# Supplementary figures and images for: COVID-19 Risk Appears to Vary Across Different Alcoholic Beverages
Source: Front Nutr. 2022 Jan 3;8:772700. doi: 10.3389/fnut.2021.772700 (PMC8761797; doi:10.3389/fnut.2021.772700)

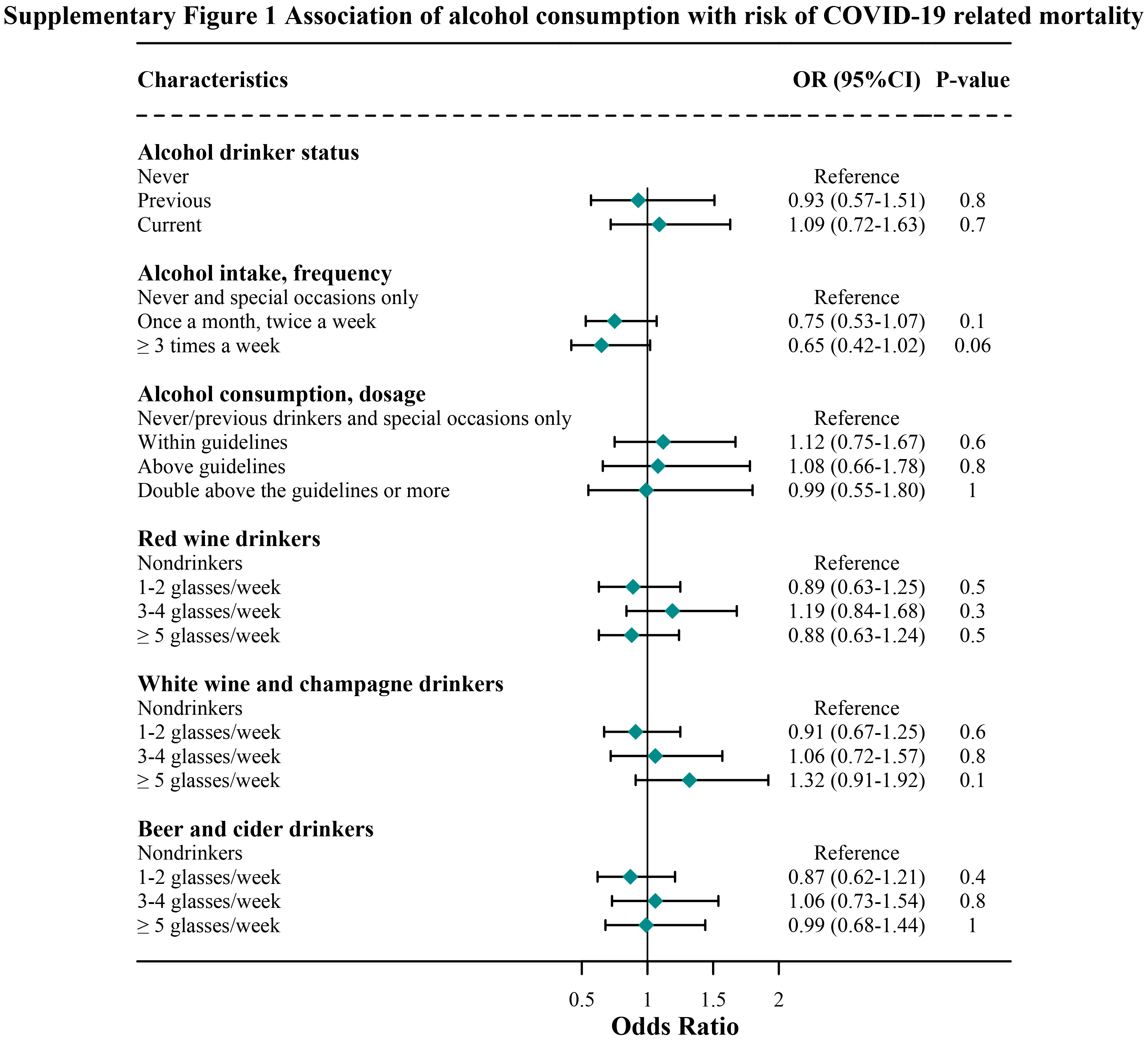

Supplement: Supplementary file 2 [file Image_1.TIF]
